# What happens when forests fall?

**DOI:** 10.7554/eLife.67863

**Published:** 2021-04-06

**Authors:** Mercedes Pascual, Andres Baeza

**Affiliations:** 1 Department of Ecology and Evolution, University of ChicagoChicagoUnited States; 2 Global Drylands Center, Arizona State UniversityChicagoUnited States

**Keywords:** malaria, *P. vivax*, deforestation, high risk population, forest-goers, greater mekong sub-region, *P. falciparum*, Other

## Abstract

Combining spatial and temporal data is helping researchers to understand how deforestation influences the risk of malaria.

**Related research article** Rerolle F, Dantzer E, Lover AA, Marshall JM, Hongvanthong B, Sturrock HJ, Bennett A. 2021. Spatio-temporal associations between deforestation and malaria incidence in Lao PDR. *eLife* **10**:e56974. doi: 10.7554/eLife.56974

Deforestation is one of the most rapid and impactful human activities on the planet. How it influences the fate of old and new pathogens is now becoming a central question for global health, especially for the populations who quickly colonize the newly cleared ‘frontier’ regions. Forests can act as reservoirs for insect species that spread deadly diseases – among them malaria, a mosquito-borne infection and one of the ‘big three’ killer illnesses in the developing world. As humans disturb forests and bring down the trees, does malaria risk increase or decrease? Deceptively simple, this question has led to contradictory answers when researchers have explored the relationship between forest cover and malaria cases (Tucker Lima et al., 2017).

Mechanistically, disturbing forests can increase exposure of vulnerable human populations to malaria (Vittor et al., 2006). It can also modify the diversity and relative success of mosquito species, favoring those that transmit malaria and increasing the incidence of the disease in frontier regions (Patz et al., 2000). However, deforestation may also lead to economic development and better living conditions, which could reduce the number of cases. And once the forest is gone, the local environment might no longer sustain the transmission cycle. As many countries aim to eliminate malaria by 2030 and tropical forests are rapidly lost, public health efforts require an understanding of how these opposite effects play out (Shretta et al., 2017; Dobson et al., 2020).

Now, in eLife, François Rerolle (University of California San Francisco; UCSF) and colleagues report new conclusions based on unprecedented data from the Lao People’s Democratic Republic (Lao PDR) in the Greater Mekong Subregion, where countries are increasingly trying to control forest malaria (Rerolle et al., 2021). The team applied a spatio-temporal statistical model to a dataset formed of longitudinal records of malaria cases in individual villages, combined with high-resolution images of forest cover obtained through remote sensing. This showed that, after deforestation, the incidence of malaria increases for a period of about two years, and then decreases. This ‘up-and-down’ trajectory can only be detected in villages located at least 30 km away from the deforestation front, and it is also more apparent for *Plasmodium falciparum* than for *Plasmodium vivax*, the two parasites responsible for malaria in the region. In other words, disease transmission gets worse before it gets better, at least for the most virulent parasite.

The work by Rerolle et al. – who are based at UCSF, UMass-Amherst, UC Berkeley and the Lao PDR Ministry of Health – helps to explain why some previous analyses had failed to identify a clear and consistent effect of deforestation: those reports focused on spatial but not temporal data, and the locations considered may have been at different stages of the transition process. Also, the data may not have examined populations at the distances from the forest front where the effects can be detected.

The study also corroborates the ‘up-and-down’ trajectory previously demonstrated for malaria in frontier regions of the Amazon (de Castro et al., 2006; Figure 1). However, the period of elevated risk was much shorter in Lao PDR. In addition, the transition for malaria caused by *P. vivax* may also be evident in some parts of the world but not others, a puzzling difference between the Amazon and forests in Lao PDR.

**Figure 1.**
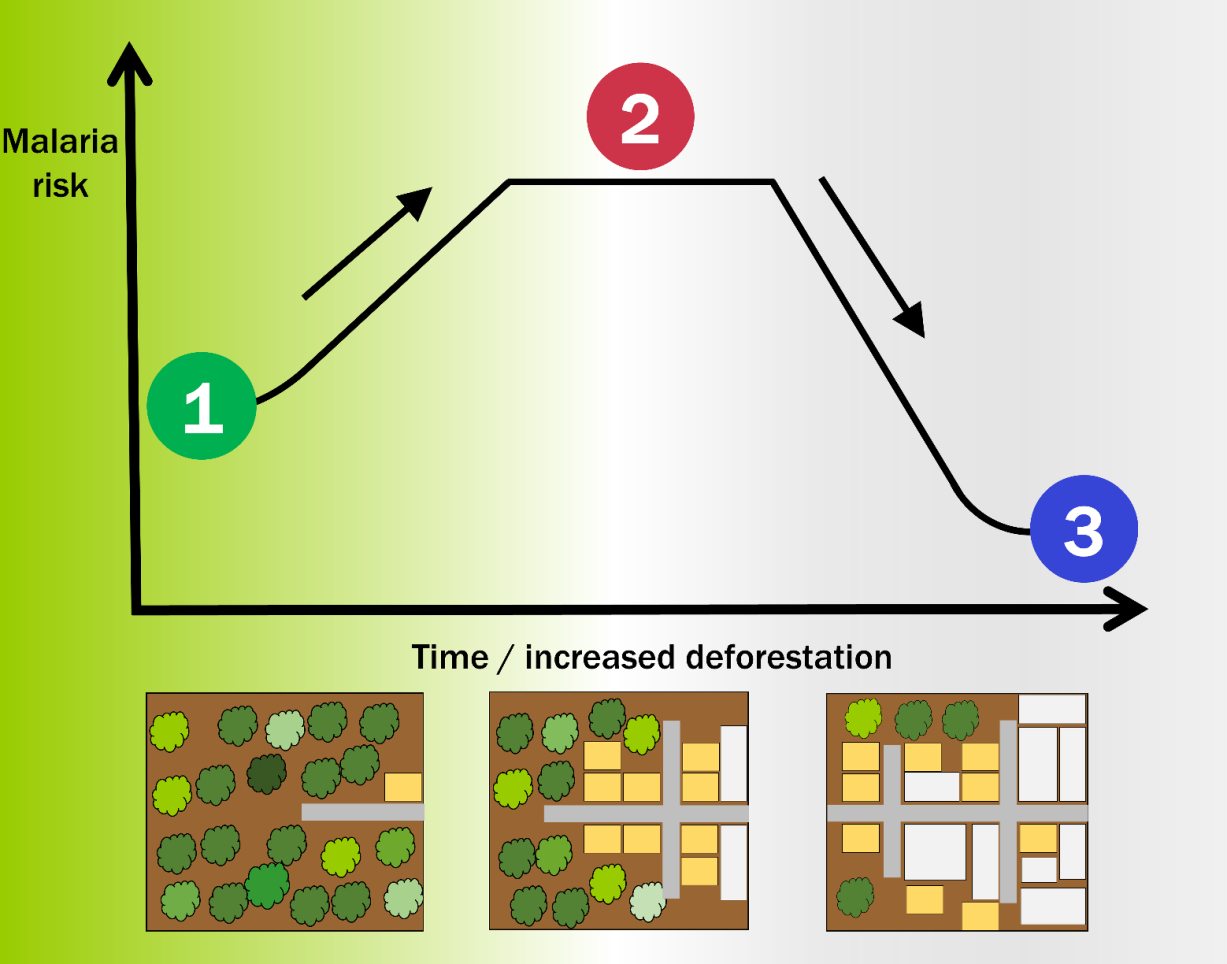
Deforestation and the incidence of malaria. Schematic diagram showing how the risk or incidence of malaria first increases and then decreases as deforestation proceeds. Before deforestation (bottom left) the forest is largely pristine, with a low population density and activities that do not cause deforestation. Malaria can be epidemic (1) and mostly driven by environmental/climatic conditions. As deforestation proceeds (bottom middle), humans start to colonize the area, roads (shown in grey) are built, and agricultural (yellow) and urban areas (white) follow. Malaria risk is enhanced (2) at this modified boundary between human settlements and the forest. Once deforestation is widespread, and after some time that depends on the region and alteration of the landscape (bottom right), the area can sustain only low but endemic malaria transmission (3); however, the risk of infection increases for other diseases transmitted by mosquitoes that thrive in this domesticated environment, such as dengue and Zika.

Beyond deforestation, other types of land-use changes can impact malaria risk in frontier regions, with, interestingly, the same temporal trajectory emerging. For instance, the arrival of irrigation in an arid region of Northwest India similarly enhanced the incidence of desert malaria due to *P. vivax*. This transition lasted at least a decade, and the disease slowed down and was nearly eliminated in parts of the region irrigated for longer (Baeza et al., 2013). Depending on when irrigation had arrived, different locations represented various stages in the temporal trajectory of the disease, creating a 'patchwork' of malaria states. In this context, understanding the impact of irrigation is impossible if the only data available are 'snapshots' with no information on when water first came to each area.

A mathematical model which combines land-use change, malaria transmission and macroeconomics helps to understand why the ‘up-and-down’ trajectories identified by Rerolle et al. and others can take place (Baeza et al., 2017). Indeed, the negative ecological effects brought by deforestation typically emerge faster than the positive economic developments that lead to better health and disease protection. Underscoring its generality, this model is consistent with the data analyses conducted for desert and forest malaria, in the Amazon and now Lao PDR. However, it does not address the consequences of losing the ecosystem services provided by a healthy forest, and the sustainable economic activities this environment sustains. In addition, while the up-and-down trajectory described by Rerolle et al. and others is the most likely to take place, the model demonstrates that it is not the only possible outcome: depending on how much is invested in the health and living conditions of the local populations after deforestation, malaria may be more prevalent than when the forest was intact (Baeza et al., 2017).

A recent bi-directional analysis of extensive malaria and forest cover data in the Amazon helps to dive further into the complexities of these interactions (MacDonald and Mordecai, 2019). The results highlighted a two-way system where deforestation and malaria influenced each other: taking down trees led to an increase in malaria cases, which in turn caused a fall in the rate of land clearing. This opens the door for the existence of a dynamical trap which involves cycles of deforestation and colonization, depending on the relative strength of each factor (Bonds et al., 2010; Ngonghala et al., 2017).

Most importantly, is one class of insect-borne disease traded for another as forests become ‘urbanized’? While malaria transitions towards low endemism after deforestation, emerging global diseases such as dengue, Zika and chikungunya are expanding their reach in the Amazon (Lowe et al., 2020). Caused by arboviruses, these new illnesses are transmitted by species of mosquitoes (notably *Aedes aegypti*) that thrive in urban environments, spreading to human settlements that have sufficiently modified the land taken from the forest. Even altered, tropical forests are not spared by arboviruses.

It is therefore clearly simplistic and dangerous to expect that, if given enough time, the economic ‘development’ enabled by land-use change will rid disturbed tropical forests of vector-borne diseases. In the case of malaria, the enhanced transmission period (whose duration depends on specific conditions) would have to be crossed, and a better outcome is not necessarily guaranteed. However, understanding how malaria unfolds in altered landscapes provides the basis for control efforts – establishing, for instance, when and where to intervene. It could also inform conservation initiatives and alternative pathways for more sustainable local economies.

At the time of COVID-19, we cannot write about tropical deforestation without underscoring that this major environmental perturbation contributes to diseases spilling from animals to humans, and promotes the emergence of new pathogens (Faust et al., 2018). Compared to the costs of preserving forests, the human and economic price of the ongoing pandemic highlights the need to urgently alter the devastating path of global deforestation (Dobson et al., 2020). From the mitigation of climate change to the prevention of emergent diseases, protecting forests is essential to safeguard human health (Gillespie et al., 2021).
